# Landscape of Somatic Alterations in Thai Pediatric Hepatoblastoma: Implications for Clinical Outcomes and Therapeutic Opportunities

**DOI:** 10.3390/medicina62040764

**Published:** 2026-04-15

**Authors:** Rinrabhat Udomwimonsit, Natakorn Nokchan, Pongsakorn Choochuen, Yanisa Klaewtanong, Surasak Sangkhathat, Kulpreeya Sirichamratsakul

**Affiliations:** 1 Department of Biomedical Sciences and Biomedical Engineering, Faculty of Medicine, Prince of Songkla University, Songkhla 90110, Thailand; rinrabhat.udo@mahidol.ac.th (R.U.); pongsakorn.c@psu.ac.th (P.C.); yanisa.klaewtanong@gmail.com (Y.K.); 2 Siriraj Center of Research Excellence for Diabetes and Obesity (SiCORE-DO), Research Department, Faculty of Medicine Siriraj Hospital, Mahidol University, Bangkok 10700, Thailand; 3 Department of Research, Faculty of Medicine Siriraj Hospital, Mahidol University, Bangkok 10700, Thailand; 4 Translational Medicine Research Center, Faculty of Medicine, Prince of Songkla University, Songkhla 90110, Thailand; natakorn.n@psu.ac.th; 5 Department of Surgery, Faculty of Medicine Siriraj Hospital, Mahidol University, Bangkok 10700, Thailand; 6 Department of Surgery, Faculty of Medicine, Prince of Songkla University, Songkhla 90110, Thailand

**Keywords:** hepatoblastoma, somatic mutation, copy number analysis, whole-exome sequencing

## Abstract

*Background and Objectives*: Hepatoblastoma (HB) is a rare pediatric liver cancer. Complete resection and chemotherapy are standard treatments, but many patients in developing countries present with unresectable tumors and show poor responses to conventional chemotherapy. Identifying somatic alterations in HB may help develop targeted molecular therapies. *Materials and Methods*: Exome sequencing was conducted on 34 HB patient samples to identify somatic mutations and copy number variations (CNVs) and to evaluate their relationships with clinical outcomes, including survival. *Results*: HB tumors showed a low mutational burden but a high rate of CNVs, averaging 181.5 CNVs compared to 3.6 somatic mutations per tumor. CNVs were enriched in pathways involved in transcription, differentiation, and development. The most common alterations were missense mutations in *KMT2D* (18%), *CTNNB1* (12%), and *MUC16* (3%). *KMT2D* mutations occurred more frequently than *CTNNB1* mutations in this cohort. Patients with *KMT2D* or *CTNNB1* mutations generally had better overall survival and longer disease-free intervals. Deletions of *ZNF429* or *FGD4* were linked to shorter survival in the cohort. Validation with an external dataset confirmed significant downregulation of *FGD4* expression in HB samples, correlating with poorer survival. *Conclusions*: This study broadens the understanding of somatic alterations in HB patients, offering insights into the molecular mechanisms behind HB development and highlighting the potential of CNV profiling and *FGD4* deletions as prognostic factors in HB.

## 1. Introduction

Hepatoblastoma (HB) is the most common primary liver cancer in children, making up about 80% of all liver tumors in those under five years old [1]. Despite being a rare embryonal malignancy with a global incidence of 0.5 to 2.0 cases per million children, this tumor has steadily increased in incidence by up to 2.7% annually in recent decades [1]. This rising trend is particularly prominent among premature and extremely low-birth-weight infants, as their prolonged exposure to oxygen therapy, growth factors, and metabolic stress during key stages of liver development might make hepatoblasts more susceptible to malignant transformation [2,3]. The clinical management of HB is challenging due to its asymptomatic early stages, frequently causing delayed diagnoses and leading 60 to 70% of patients to present with advanced disease [3,4]. This delay alongside rapid tumor growth and early vascular involvement renders immediate curative surgery unfeasible for most presenting cases [3]. Current treatment, therefore, relies on risk-stratified protocols combining neoadjuvant chemotherapy with delayed surgery [5,6]. Standard regimens such as cisplatin and doxorubicin achieve event-free survival rates exceeding 85% in low- to intermediate-risk patients [2,5]. However, outcomes remain poor in high-risk groups, particularly those with metastases, multifocal disease, or vascular invasion, with five-year overall survival declining to 60 to 70% [7].

The molecular characterization of HB has uncovered a surprisingly simple genomic landscape compared to other pediatric solid tumors, with whole-exome and whole-genome sequencing studies consistently showing a very low mutational burden, averaging 2–5 somatic mutations per tumor [8,9,10,11,12]. This scarcity of genetic alterations distinguishes HB from other pediatric cancers and suggests that only a few key oncogenic events may be sufficient to cause hepatoblast transformation. The Wnt/β-catenin signaling pathway is the primary molecular pathway involved in HB development, with *CTNNB1* mutations being the most common genetic change, present in 60–90% of cases across various studies and populations [13,14]. These mutations typically affect exon 3 of *CTNNB1*, targeting critical phosphorylation sites (Ser33, Ser37, Thr41, Ser45) within the glycogen synthase kinase-3β (*GSK-3β*) recognition sequence, resulting in stabilization of β-catenin protein and continuous activation of the Wnt pathway [14,15]. In addition to *CTNNB1*, HB’s molecular diversity involves alterations in other cancer-related genes such as *AXIN1*, *APC,* and *TP53*, although these are less frequent, occurring in 5–15% of cases [8]. Notably, when present, TP53 mutations are associated with more aggressive disease and worse clinical outcomes, highlighting their potential as prognostic biomarkers [9]. Recent extensive genomic studies have also identified recurrent chromosomal alterations, including gains in chromosomes 2q, 8q, and 20, which may facilitate oncogene activation and tumor growth [16].

Experimental evidence from Bowen and Monga shows that β-catenin activation alone is not sufficient to induce HB formation in mouse models, indicating that additional oncogenic hits or specific developmental contexts are necessary [17]. Furthermore, the molecular basis of chemotherapy resistance in high-risk HB remains poorly understood, which hinders efforts to develop strategies to overcome treatment failure. Identifying predictive biomarkers for therapeutic response, patient stratification, and prognosis is a critical unmet need in the field.

The primary objective of this study is to characterize the landscape of somatic alterations, including mutations and copy number variations, in Thai pediatric hepatoblastoma. Secondary exploratory analyses aim to assess potential associations between genomic alterations and clinical features, and to identify candidate biomarkers or therapeutic targets. Through this approach, we seek to provide population-specific genomic insights that may contribute to improved understanding and future risk stratification in hepatoblastoma. The findings may ultimately lead to the development of precision medicine approaches that improve clinical outcomes and reduce treatment-related toxicities in pediatric HB patients.

## 2. Materials and Methods

### 2.1. Sample Collection

Tumor samples were collected from 34 histologically confirmed HB patients who had received previous platinum-based chemotherapy between 2004 and 2020 at Songklanagarind Hospital. Corresponding normal adjacent tissues or blood samples were unavailable in these cases. Each tumor sample was immediately snap-frozen in liquid nitrogen after surgical removal and stored at −80 °C in the biological repository of the Translational Medicine Research Center, Faculty of Medicine, Prince of Songkla University (Songkhla, Thailand) until analysis. Informed consent was obtained from the guardians of all patients prior to participation, allowing the collection of clinical data, the acquisition and storage of biological samples, the experimental analysis, and the publication of relevant study results. Patients with incomplete medical records or missing CT data needed to determine PRETEXT and/or POSTTEXT staging were excluded from the study. The clinical data analyzed—including age at diagnosis, sex, birth weight, serum alfa-fetoprotein (AFP) level, and radiological parameters such as initial tumor size, PRETEXT, and/or POSTTEXT staging—were collected from electronic medical records at Songklanagarind Hospital. This study was approved by the Ethical Committee of the Faculty of Medicine, Prince of Songkla University (REC 62-386-10-1).

### 2.2. Whole-Exome Sequencing (WES) Sequencing

DNA extraction was performed using a Qiagen DNA Mini kit (Qiagen, Hilden, Germany). The quality of each tumor specimen’s DNA was assessed using a NanoDrop 2000 spectrophotometer (Thermo Scientific, Wilmington, DE, USA) and agarose gel electrophoresis. The human exonic regions were captured with an Agilent SureSelect XT Human All Exon v5 kit (Agilent Technologies, Santa Clara, CA, USA). Subsequently, the sequence library was constructed using the SureSelect XT Target Enrichment System for Illumina Paired-End Sequencing Library kit (Agilent Technologies, Santa Clara, CA, USA), following the manufacturer’s instructions. The library preparations were sequenced on the Illumina NovaSeq 6000 platform (Illumina, San Diego, CA, USA), producing 150 bp paired-end reads with an average target depth of 100× coverage.

The FASTQ files from WES were processed with Trimmomatic to trim adapters and remove low-quality reads (quality score below 15 or read length less than 36 bp). Only high-quality reads were aligned to the human reference genome (GRCh38) with BWA-MEM (version 0.7.12) using default settings. The resulting Sequence Alignment Map (SAM) files were converted to Binary Alignment Map (BAM) format and sorted with SAMtools (version 1.17). The sorted BAM files were then regrouped, and duplicate sequences were marked with Picard (version 3.0.0). To recalibrate base quality scores, non-duplicate BAM files were processed using GATK (version 4.4.0). Finally, the aligned reads were stored in BAM format for subsequent somatic mutation and copy number variant calling.

### 2.3. Panel of Normals (PoN) Construction

Because the study involved only tumor samples, creating a Panel of Normals (PoN) was a crucial step to minimize false-positive somatic mutations arising from germline variants or sample-preparation artifacts [18]. Fifty blood-derived WES datasets were randomly selected from the NCBI BioProject PRJNA795330 in FASTQ format using the SRA Toolkit (v3.1.0) (accessed on 20 October 2024), and these datasets served as normal samples for PoN construction. Clinical information of the patients included in the PoN, along with their average read depths, is provided in Supplementary Table S1. All blood WES samples were processed with the same pipeline as the tumor samples. Mutect2 was then run in tumor-only mode on each normal sample, and the resulting VCF files were merged using CreateSomaticPanelOfNormals.

### 2.4. Somatic Mutation Calling

Somatic single-nucleotide variants (SNVs) and small indels were identified from each tumor BAM file using GATK4 (v4.4.0.0) Mutect2 with default parameters in tumor-only mode. This mode was run with the PoN VCF file to filter out false-positive somatic calls. The variants were further filtered for contamination fractions using FilterMutectCalls in GATK4, and only somatic variants with read depth (DP) > 10 that passed filtering were retained. The remaining mutations were annotated against the Catalogue of Somatic Mutations in Cancer (COSMIC) database using OpenCRAVAT (https://run.opencravat.org, accessed on 19 November 2024). The annotated mutations were stored in MAF files, which were then summarized into mutation profiles and visualized with the maftools package in R (v4.3.0). Cancer-related genes were identified by filtering variants with a customized cancer-associated gene panel (905 genes, Supplementary Table S2), derived from public resources including the Cancer Gene Consensus from the COSMIC v101 database for GRCh38 and the Pediatric Cancer Variant Pathogenicity Information Exchange (PeCanPIE) [19,20].

### 2.5. Mutational Signatures Analysis

Somatic mutations in cancer cells originate from various mutational processes, including both internal mechanisms and external exposures. Each process produces characteristic combinations of mutation types, known as mutational signatures, which are important for cancer development. In this study, mutational signature fitting was performed using SigProfiler (https://cancer.sanger.ac.uk/signatures/assignment/, accessed on 23 November 2024) with default settings. Mutational profiles were obtained from MAF files and then refitted to COSMIC version 3.4, which includes 86 single-base substitution (SBS) signatures, to determine the contribution of known mutational processes [21]. Results were displayed using SigProfilerPlotting module within SigProfiler with default options.

### 2.6. Identification of Cancer Driver Genes and Biomarkers for Therapeutic Targets and Drug Response

To annotate driver mutations that confer growth advantages to cancer cells and promote HB initiation and progression, all annotated somatic mutations from the MAF files were analyzed using the Cancer Genome Interpreter (CGI; https://www.cancergenomeinterpreter.org, accessed on 3 February 2025). Two web-based machine learning tools, BoostDM and OncodriveMUT, were applied to identify potential driver mutations and biomarkers for anticancer therapies and drug responses, with HB selected as the cancer type for annotation. Both methods enabled categorization of variants into annotated (known), predicted drivers, and passenger mutations according to the Catalog of Validated Oncogenic Mutations.

### 2.7. Copy Number Variants (CNVs) Calling

Copy number variants were analyzed using CNVkit (v0.9.4) by comparing tumor BAM files against a normal reference built from 50 blood WES samples. Log_2_ copy number ratios were generated and segmented with the circular binary segmentation (CBS) algorithm. Segmented ratios were normalized using average tumor ploidy values estimated by PureCN (v2.0.1) prior to CNV calling, with tumor purity applied as the threshold. Significantly amplified and deleted regions at both the chromosome arm and gene levels were identified using GISTIC (v2.0) through the GenePattern web interface (https://www.genepattern.org/, accessed on 22 November 2024) with default parameters. Data visualization was performed using the maftools and ggplot2 R packages. Copy number states were defined as follows: deletion (CN = −2), loss (CN = −1), normal (CN = 0), gain (CN = 1), and amplification (CN = 2).

### 2.8. Functional and Pathway Enrichment Analysis

Gene Ontology (GO) enrichment analysis was conducted using the DAVID online tool (https://davidbioinformatics.nih.gov/, accessed on 3 February 2025) to examine the biological functions of genes affected by somatic mutations and CNV alterations in HB. GO terms were divided into three domains: Biological Process (BP), Cellular Component (CC), and Molecular Function (MF). Additionally, the KEGG and Reactome pathway databases were used to explore the involvement of genes harboring somatic mutations and CNVs in known signaling and metabolic pathways. DAVID was also employed to analyze KEGG and Reactome enrichment, excluding pathways with fewer than 10 genes. A q-value < 0.05 was considered indicative of significant enrichment. The findings were visualized using the ggplot2 package in R.

### 2.9. Survival Analysis

Overall survival (OS) was defined as the time from initial diagnosis to death or last follow-up, while disease-free survival (DFS) was defined as the time from diagnosis to recurrence, disease progression, death, or last contact, whichever occurred first. Patients were grouped according to genomic alterations based on mutation status (mutant versus wild-type) or CNV status (amplification versus deletion). Kaplan–Meier curves were used to estimate median OS and DFS, and differences between groups were analyzed with the log-rank test. The relationships between clinicopathological variables and genomic alteration status were examined using the chi-squared or Fisher’s exact test, as appropriate. Statistical significance was set at a *p*-value < 0.05.

### 2.10. Validating the Expression and Prognosis Value of CNV

To verify CNV-driven genes related to HB survival, RNA expression datasets were obtained from the R2 Genomics Analysis and Visualization Platform (https://hgserver1.amc.nl/, accessed on 26 February 2025), including Raymond (GSE104766), Carrillo-Reixach (GSE133039), Kappler (GSE151347), Ikeda (GSE131329), and López-Terrada (GSE75271). Additionally, gene expression and survival data from 583 hepatocellular carcinoma (HCC) patients were collected from the Human Protein Atlas database (v22.0; https://www.proteinatlas.org/, accessed on 26 February 2025) for survival analysis.

## 3. Results

### 3.1. Baseline Characteristics of the 34 Patients with HB

A total of 34 HB patients were included in this study, and their clinicopathological characteristics are shown in Table 1. The cohort comprised 23 males and 11 females, with ages ranging from 1 month to 11 years and 6 months. HB is generally observed in children under 3 years old, with a male predominance. Based on pre-treatment clinical imaging studies, the PRETEXT system classified 14 HB cases (41.2%) as stage II, 13 (38.2%) as stage III, and 7 (20.6%) as stage IV. Treatment for all HB patients followed risk-stratification guidelines according to the Thai Pediatric Oncology Group (ThaiPOG) protocol, with 6 (17.6%) patients classified as low risk, 12 (35.3%) as intermediate risk, and 16 (47.1%) as high risk. The median serum AFP level at diagnosis was 57,335 ng/mL (range, 1–800,464 ng/mL). Only 3 patients (8.8%) had a serum AFP level below 100 ng/mL at diagnosis, a level correlated with a poor prognosis.

### 3.2. Mutation Profiles of the 34 Patients with HB

**Figure 1:**
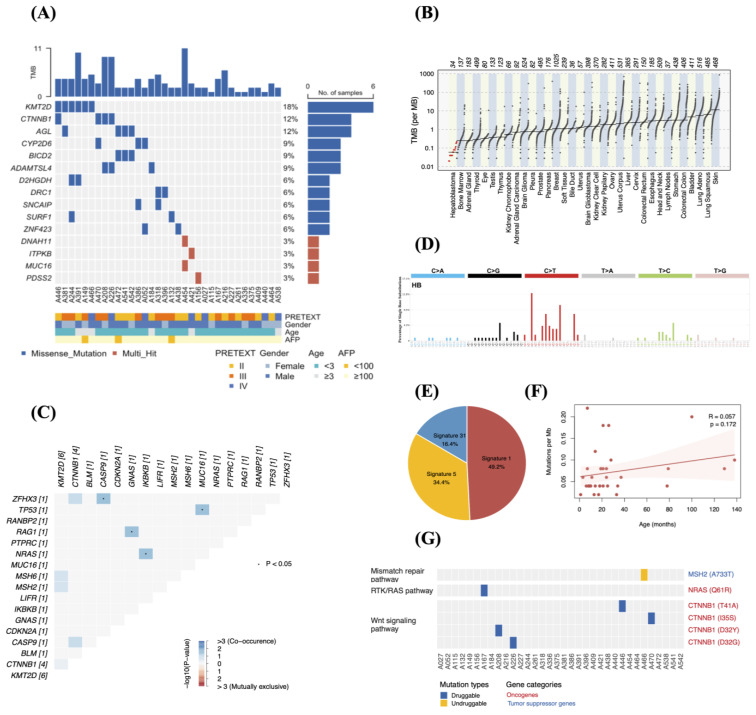
The genomic landscape and mutational features of HB. (**A**) Distribution of somatic mutations in 34 HB cases. (**B**) Comparison of tumor mutation burden (TMB) across TCGA datasets, with HB samples highlighted in red. (**C**) Somatic interactions among cancer-related genes, with co-occurrence shown in blue, mutual exclusivity in red, and significant events marked by dots (*p* < 0.05). (**D**) Mutational signatures derived from the HB mutation catalog. (**E**) Relative contributions of each signature. (**F**) Correlation between age at diagnosis and TMB, evaluated using Spearman correlation. (**G**) Distribution of driver mutations in 34 HB cases, categorized as druggable (blue) or undruggable (yellow), with oncogenes labeled in red and tumor suppressor genes in blue.

To identify the genomic alterations in HB tumors, we conducted whole-exome sequencing (WES) on samples from 34 patients. Raw sequencing reads (FASTQ files) were processed with Trimmomatic to eliminate Illumina adapters, low-quality bases (Phred score < 15), and short reads (<36 bp), then aligned to the human reference genome (GRCh38) using BWA. The average sequencing depth in targeted regions was approximately 102.25×. A total of 123 somatic mutations across 94 genes were detected, averaging 3.6 mutations per patient (0.06 mutations/MB). The 15 most frequently mutated genes include *KMT2D*, *CTNNB1*, *AGL*, *CYP2D6*, *BICD2*, *ADAMTSL4*, *D2HGDH*, *DRC1*, *SNCAIP*, *SURF1*, *ZNF423*, *DNAH11*, *ITPKB*, *MUC16*, and *PDSS2* (Figure 1A). We also compared the tumor mutational burden (TMB) in HB with that of 32 different cancer types from The Cancer Genome Atlas (TCGA) database. This analysis showed that HB has the lowest TMB among all cancer types examined (Figure 1B). Furthermore, the study identified mutations in 17 cancer-related genes known to be involved in pediatric cancer. These include *KMT2D*, *CTNNB1*, *MUC16*, *RAG1*, *BLM*, *CASP9*, *CDKN2A*, *GNAS*, *IKBKB*, *LIFR*, *MSH2*, *MSH6*, *NRAS*, *PTPRC*, *RANBP2*, *TP53*, and *ZFHX3*. Notably, missense mutations in *KMT2D*, *CTNNB1*, and *MUC16* were among the most commonly observed alterations.

To analyze patterns of co-mutation and mutual exclusivity, we conducted pairwise overlap analysis of the identified cancer-associated genes in HB using Fisher’s exact test. Significant pairwise interactions are shown in Figure 1C. Notably, mutations in the oncogene *MUC16* co-occurred exclusively with *TP53* mutations.

### 3.3. Mutational Signatures Reveal Chemotherapy-Associated DNA Damage in HB

Our analysis of somatic base substitution patterns in the WGS data from 34 HB genomes (Figure 1D) showed that the most common base substitution was the C-to-T transition (57.4%), followed by the T-to-C transition (16.4%) and the C-to-G transversion (15.6%). These substitution patterns were broken down into known mutational signatures from COSMIC, revealing contributions from Signature 1 (49.2%), Signature 5 (34.4%), and Signature 31 (16.4%) (Figure 1E). Signature 1 and Signature 5 were linked to aging and were found in various adult and pediatric cancers [22]. However, the tumor mutational burden (TMB) of HB was not significantly related to age at diagnosis (Spearman correlation coefficient = 0.018, *p* = 0.509) (Figure 1F). Additionally, all samples had prior exposure to platinum-based chemotherapy, and the presence of SBS31 is consistent with treatment-associated mutagenesis. This indicates that a substantial proportion of the observed mutations likely reflects chemotherapy-induced DNA damage and repair mechanisms rather than intrinsic tumor biology [23].

### 3.4. Identification of Driver Mutations and Potential Therapeutic Targets in HB

We used the Cancer Genome Interpreter (CGI) to assess the 123 unique variants identified in this study. The analysis revealed driver mutations in three genes: *MSH2*, *NRAS*, and *CTNNB1*, as shown in Figure 1G. Among these, driver mutations in *CTNNB1* were the most frequently detected in HB, resulting in the absence of β-catenin degradation (22). This leads to aberrant activation of the Wnt pathway, promoting uncontrolled cell proliferation and tumor formation in HB and various cancers (23). CGI was also used to identify potential drug targets for the detected driver mutations. The analysis indicated druggable mutations in two cancer driver genes, *NRAS* and *CTNNB1*, as summarized in Supplementary Table S3. Although the efficacy of platinum-based agents in HB cases with these mutations has not been evaluated, previous clinical trials have demonstrated favorable responses to chemotherapeutic agents in cutaneous melanoma with *NRAS* (Q61R) mutations, which occur in approximately 3% of HB cases. Moreover, preclinical studies have suggested that *CTNNB1* (T41A, I35S, D32Y, and D32G) mutations may serve as biomarkers of resistance to tyrosine kinase inhibitors in colorectal cancer. These findings should therefore be interpreted with caution, and further functional and clinical studies in HB are required to determine their therapeutic relevance.

### 3.5. CNV Profiles of the 34 Patients with HB

**Figure 2:**
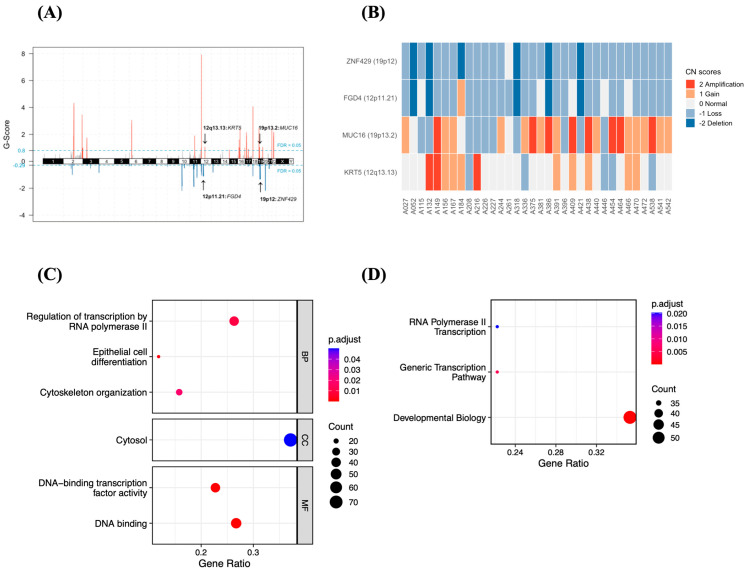
Somatic copy number variation (CNV) profiles and pathway enrichment in HB. (**A**) Significant focal amplifications (red) and deletions (blue) across 34 HB cases, with the blue line indicating the FDR threshold of 0.05. (**B**) Distribution of CNVs in cancer-associated genes. (**C**) Gene Ontology (GO) enrichment analysis and (**D**) KEGG/Reactome pathway analysis of 301 genes located within CNV regions.

GISTIC analysis of 34 HB samples revealed significant chromosomal abnormalities involving amplifications and deletions, both over the chromosome arm and at the cancer-associated gene. Broad regions of amplification were observed on chromosome arms 9p, 11p, 20p, 20q, and 22q, while deletions were identified on 4q, 7p, 7q, 19p, 19q, and 22p (Supplementary Table S4). GISTIC further identified focal peaks in 39 cytoband regions covering 301 genes, including 18 amplified and 21 deleted regions, as shown in Figure 2A and Supplementary Table S5. On average, each tumor exhibited 181.5 focal copy number changes, ranging from 125 to 242. The most frequently amplified region was 19p13.2, occurring in 23.5% of samples and targeting *MUC16*, followed by 12q13.13 (8.8%), which targets *KRT5*. The most frequently deleted region was 19p12, found in 17.6% of samples and affecting the cancer-associated gene *ZNF429*. Another deletion involved chromosomes 12p11.21 (14.7%), which contains the known cancer gene *FGD4* (Figure 2B).

Additionally, we conducted enrichment and pathway analyses to evaluate the functions of the 301 genes identified in 39 significant CNV segments. The GO analysis revealed that these genes are involved in transcriptional regulation by RNA polymerase II, epithelial cell differentiation, cytosol, and DNA binding (Figure 2C). KEGG and Reactome analyses showed that CNV-related genes are enriched in pathways associated with transcriptional regulation and developmental biology (Figure 2D). Since most of these enriched pathways are associated with transcription, differentiation, and development, this supports previous studies suggesting that dysregulation of transcriptional pathways during fetal liver development may play a key role in the occurrence of HB [24].

### 3.6. Genomic Alterations in HB and Their Clinical Relevance

**Figure 3:**
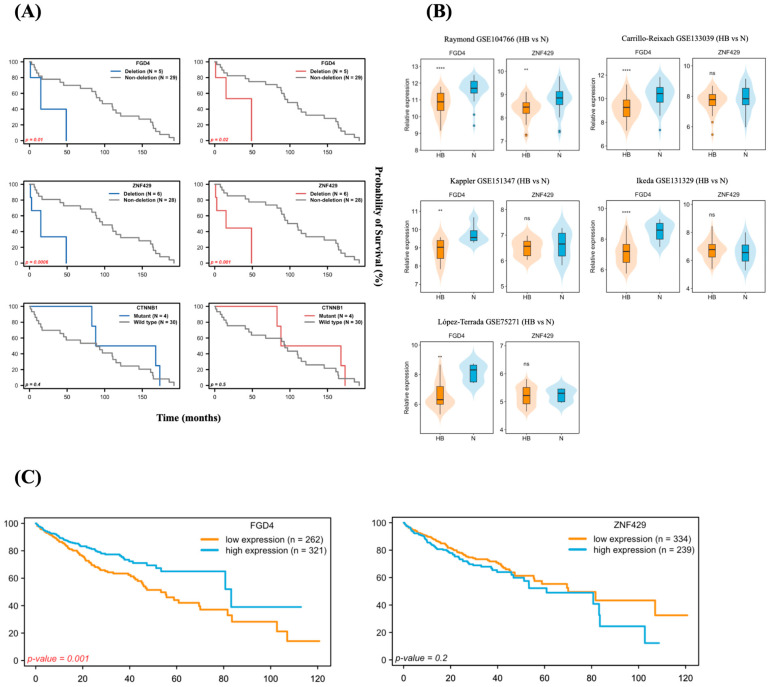
Prognostic impact of CNV-associated genes and their expression. (**A**) Kaplan–Meier survival curves showing disease-free survival (DFS, blue) and overall survival (OS, red) in 34 HB patients, comparing cases with and without *FGD4* deletion (*n* = 5 vs. *n* = 29), *ZNF429* deletion (*n* = 6 vs. *n* = 28), and *CTNNB1* mutations (*n* = 4 vs. *n* = 30). Significance was determined by the log-rank test. (**B**) RNA expression levels of *FGD4* and *ZNF429* in HB versus normal liver (N) across five independent GEO datasets: Raymond GSE104766 (HB = 30, *n* = 30), Carrillo-Reixach GSE133039 (HB = 31, *n* = 32), Kappler GSE151347 (HB = 11, *n* = 11), Ikeda GSE131329 (HB = 53, *n* = 14), and López-Terrada GSE75271 (HB = 50, *n* = 5). Student *t*-test was applied to determine statistical significance, where ** *p* < 0.01 and **** *p* < 0.0001 (**C**) Kaplan–Meier survival curves showing overall survival in HCC patients (*n* = 583), stratified by high versus low expression of *FGD4* and *ZNF429*, retrieved from the Human Protein Atlas. Significance was determined by the log-rank test.

Cancer-associated genes among the 15 most frequent somatic mutations and CNV profiles were selected for assessment of clinical significance (Supplementary Figure S1). Kaplan–Meier curves showed notable differences in DFS and OS between patients with and without deletions in *FGD4* and *ZNF429* (Figure 3A). Patients with *FGD4* deletions experienced significantly worse outcomes, with a median DFS of 14.9 vs. 96.9 months (*p* = 0.01) and a median OS of 48.8 vs. 96.9 months (*p* = 0.02). Likewise, *ZNF429* deletions were associated with poorer survival, with median DFS of 14.9 vs. 96.9 months (*p* = 0.0006) and median OS of 14.9 vs. 110.0 months (*p* = 0.001). We also examined the relationships between clinicopathological variables and deletions of *FGD4* and *ZNF429*, as detailed in Supplementary Table S6. No significant associations were found between these deletions and any clinicopathological factors in HB patients.

Additionally, we focused on *CTNNB1*, the most frequently altered driver gene in this study (4 of 34 cases; 11.8%). Among all HB patients, those with *CTNNB1* mutations (*n* = 4) appeared to respond better to platinum-based chemotherapy and showed a trend toward improved outcomes. The median OS was 128 months in the *CTNNB1-mutant* group versus 93 months in the wild-type group (*n* = 30), although this difference was not statistically significant (*p* = 0.5). Similarly, the median disease-free survival (DFS) was 128 months for patients with *CTNNB1* mutations and 89 months for those without (*p* = 0.4), as shown in Figure 3A. Overall, these findings suggest that deletions of *FGD4* or *ZNF429* are significantly linked to shorter OS and DFS. Moreover, although the differences in OS and DFS between patients with and without *CTNNB1* mutations were not statistically significant, there was a trend toward better survival and longer disease-free periods in HB patients with *CTNNB1* mutations. However, given the small sizes of the specific alteration subgroups, these survival analyses should be considered exploratory, and the statistical limitations associated with small cohorts should be carefully considered.

### 3.7. FGD4 Acts as a Potential CNV-Driven Gene Associated with Poor Prognosis in Liver Cancer

Several studies have shown that somatic CNVs can promote the development and progression of various cancers by disrupting gene function or altering gene expression [25,26,27,28]. CNV-driven genes were identified based on consistent patterns between significant copy number changes and differential gene expression [26,28]. To examine the expression levels of deleted *FGD4* and *ZNF429*, we retrieved data from five publicly available HB datasets (totaling 175 cases) in the R2 database (Figure 3B). We found that *FGD4* expression was significantly lower in HB samples compared to normal liver tissue across all datasets. However, *ZNF429* showed significantly lower expression in only one dataset (Raymond GSE104766; HB = 30 cases).

Next, we evaluated the survival probability of liver cancer patients with high and low expression levels of *FGD4* and *ZNF429* genes. Due to the limited availability of survival data for patients with HB, we analyzed data from patients with hepatocellular carcinoma (HCC; *n* = 583) obtained from the Human Protein Atlas database. Expression levels were measured for each case and divided into high- and low-expression groups using the mean expression level of each gene as the cutoff. Kaplan–Meier analysis revealed that patients with low *FGD4* expression had significantly worse OS than those with high expression (Figure 3C), with median OS of 53.3 versus 83.2 months (*p* = 0.001). The 5-year survival rate was also lower in the low-expression group (44.0% versus 65.0%). For *ZNF429*, the difference in OS between low- and high-expression groups was not statistically significant (Figure 3C), with median OS of 70.0 versus 60.8 months (*p* = 0.2) and estimated 5-year survival rates of 55.4% versus 52.3%. In conclusion, our findings suggest that *FGD4* may serve as a CNV-driven gene in HB. Additionally, deletion of *FGD4* is associated with poor prognosis in both HB and liver cancer.

## 4. Discussion

Our detailed genomic analysis of Thai HB patients shows a unique molecular profile characterized by a very low mutation burden, averaging only 3.6 somatic mutations per tumor, paired with an extremely high rate of CNVs, averaging 181.5 per tumor. This genomic pattern indicates that HB development is primarily driven by large-scale chromosomal changes rather than point mutations, distinguishing it from many adult cancers, which typically have higher mutational loads [29]. The predominant mutational signatures identified in our cohort (COSMIC Signatures 1, 5, and 31) provide important insights into the etiology of observed mutations. These signatures are consistent with chemotherapy-induced DNA damage and subsequent repair processes, suggesting that a significant portion of the mutational landscape may be iatrogenic rather than tumor-initiating [23]. This finding has important implications for understanding the temporal evolution of HB genomics and highlights the need for careful interpretation of mutations detected in post-treatment samples. The prevalence of these treatment-associated signatures underscores the importance of analyzing pre-treatment samples whenever possible to distinguish driver mutations from therapy-induced alterations.

Our analysis revealed differences in the frequency of key driver mutations. *KMT2D* mutations were the most common, identified in 18% of cases, and occurred more frequently in our Thai cohort than *CTNNB1* mutations, which were found in 12% of cases. This differs from reports in Western populations, where *CTNNB1* mutations typically predominate. A study by Liu et al. reported similar findings in an Asian cohort, including a relatively low *CTNNB1* mutation rate (12.5%) and a higher frequency of *KMT2D* mutations (25%) [30]. Importantly, they also demonstrated that *KMT2D* knockdown significantly inhibited HB cell growth, supporting its functional role in tumor development. These findings are consistent with our results and suggest that alternative driver events, such as *KMT2D* alterations, may contribute to tumorigenesis in certain populations. The *KMT2D* gene encodes a histone methyltransferase responsible for methylating lysine 4 on histone H3 (H3K4), a modification associated with active gene transcription [31]. Mutations in *KMT2D* have been reported in multiple cancers and are associated with favorable outcomes in pancreatic and breast cancers [32,33]. However, given the relatively small sample size, this observation should be considered a hypothesis. Further validation in larger, independent Asian cohorts is required to confirm this potential population-associated pattern. Although less frequent in our cohort, *CTNNB1* mutations remain clinically important as they activate the Wnt pathway, a key oncogenic driver in HB. Our data indicated a trend toward better responses to platinum-based chemotherapy among patients with CTNNB1 mutations. However, these findings should be considered exploratory given the small sample size. This observation aligns with emerging evidence that Wnt pathway alterations may improve chemosensitivity through mechanisms involving DNA repair pathways. Furthermore, regarding the potential therapeutic targets identified by the Cancer Genome Interpreter (such as *NRAS* and *CTNNB1*), it is crucial to emphasize that the proposed associations between these mutations and drug responses are primarily based on studies in other tumor types, such as melanoma and colorectal cancer. Because oncogenic signaling and drug efficacy are highly tissue-specific and context-dependent, these specific vulnerabilities cannot be directly extrapolated to hepatoblastoma. Therefore, any direct inference of clinical relevance in HB is currently premature. These potential therapeutic avenues remain strictly theoretical and require extensive preclinical testing and validation, particularly in HB models, before any clinical application can be considered.

The high prevalence of CNVs in our cohort, particularly deletions of *FGD4* and *ZNF429*, represents a paradigm shift in understanding HB pathogenesis. These non-mutation-based mechanisms may represent important drivers of HB growth and progression. *FGD4*, encoding an F-actin-binding protein essential for cytoskeletal organization, showed significant downregulation in HB samples [34]. Previous studies in prostate cancer have demonstrated that *FGD4* loss reduces cell proliferation and migration while altering drug sensitivity [34]. In contrast, our findings reveal that *FGD4* deletions and its significant downregulation are strongly linked to shorter survival in HB, likely by disrupting normal cell polarization and thereby promoting an aggressive, metastatic phenotype. The specific role of *FGD4* in cell polarization and metastatic signaling will be the subject of detailed functional investigations in future studies. *ZNF429*, a zinc-finger transcription factor involved in cellular differentiation and developmental gene regulation, is another critical target of chromosomal deletion [35,36]. The loss of these regulatory proteins likely disrupts normal hepatocyte maturation programs, contributing to the undifferentiated phenotype characteristic of HB. The co-occurrence of *FGD4* and *ZNF429* deletions in many tumors suggests these alterations may cooperate in HB pathogenesis.

Most notably, deletions of *ZNF429* or *FGD4* were associated with shorter overall and disease-free survival, suggesting their potential as prognostic biomarkers. Due to the limited access to publicly available pediatric HB survival data, these markers were evaluated using hepatocellular carcinoma (HCC) datasets. This surrogate approach is consistent with recent pediatric liver cancer research, as HCC also occurs in pediatric patients and shares certain underlying molecular properties with HB [36]. These findings provide preliminary evidence of an aggressive tumor biology that could refine future risk stratification. Specifically, patients harboring these deletions might benefit from more intensive surveillance, alternative treatment plans, or participation in clinical trials exploring new therapies. However, given the limited sample size, further functional studies and validation in larger, independent HB cohorts remain essential to confirm the clinical utility of these markers in precision therapeutic decision-making.

Several limitations should be acknowledged. The relatively small cohort size limits the power to detect rare alterations and may not fully capture the genomic diversity of Thai HB patients. Furthermore, the limited sample size restricts the scope of bioinformatics analyses and may preclude statistically significant findings. A major methodological limitation is the absence of matched normal samples, which restricts the ability to distinguish somatic from germline variants. Although a panel of normals and stringent filtering criteria were applied, tumor-only analysis remains more susceptible to germline false positives, and the results should therefore be interpreted with caution. In addition, the exclusive use of post-treatment samples represents a major confounding factor in this study. The detection of chemotherapy-associated mutational signatures, particularly SBS31, indicates that treatment-induced DNA damage has substantially influenced the observed genomic landscape. This may mask or alter the underlying driver events and limit the ability to fully interpret the intrinsic biology of this tumor. Finally, the use of hepatocellular carcinoma survival data as a surrogate for HB outcomes, while necessary due to limited data availability, requires validation in larger pediatric HB cohorts.

## 5. Conclusions

In conclusion, this study demonstrates that hepatoblastoma in Thai patients has a distinctive genomic profile characterized more by copy number alterations than by point mutations, with unique ethnic-specific patterns in driver gene involvement. The discovery of *FGD4* and *ZNF429* deletions as prognostic biomarkers represents a significant advancement in HB molecular classification and risk assessment. The identification of treatment-responsive molecular subtypes, especially *CTNNB1*-mutated tumors, indicates that genomic profiling should be incorporated into clinical decision-making. These findings provide a foundation for developing personalized medicine approaches tailored to the specific molecular characteristics of HB across different populations.

## Figures and Tables

**Table 1:** The Clinicopathological characteristics of 34 patients with HB who were included in this study.

Variables	Category	Number of Cases (%)
Age (years)	<3	28 (82.4)
	>3	6 (17.6)
Gender	Male	23 (67.6)
	Female	11 (32.4)
PRETEXT stage	II	14 (41.2)
	III	13 (38.2)
	IV	7 (20.6)
Risk stratification	Low	6 (17.6)
	Intermediate	12 (35.3)
	High	16 (47.1)
Pretreatment AFP	<100 ng/mL	3 (8.8)
	>100 ng/mL	31 (91.2)

## Data Availability

The data presented in this study are available on request from the corresponding author due to ethical concerns regarding patient privacy.

## Supplementary Materials

The following supporting information can be downloaded at: https://www.mdpi.com/article/10.3390/medicina62040764/s1, Supplementary Figure S1: Kaplan–Meier analyses of disease-free survival (DFS) and overall survival (OS) for cancer-associated genes among the 15 most frequently observed somatic mutations and CNV profiles in HB; Supplementary Table S1: The clinical information of the patients selected to create the PoN; Supplementary Table S2: List of our customized cancer-associated genes; Supplementary Table S3: The potential targeted drugs for the driver mutations in HB; Supplementary Table S4: CNV profiles at chromosome arm identified in 34 HB patients; Supplementary Table S5: CNV profiles of cancer-associated genes in focal regions identified in 34 HB patients; Supplementary Table S6: Association between clinical variables and deletion of *FGD4* and *ZNF429*.
