# Supplementary material for: Landscape of Somatic Alterations in Thai Pediatric Hepatoblastoma: Implications for Clinical Outcomes and Therapeutic Opportunities
Source: Medicina (Kaunas). 2026 Apr 15;62(4):764. doi: 10.3390/medicina62040764 (PMC13117025; doi:10.3390/medicina62040764)
Supplement: Supplementary file 1 [file medicina-62-00764-s001.zip › medicina-4233458-supplementary.pdf]

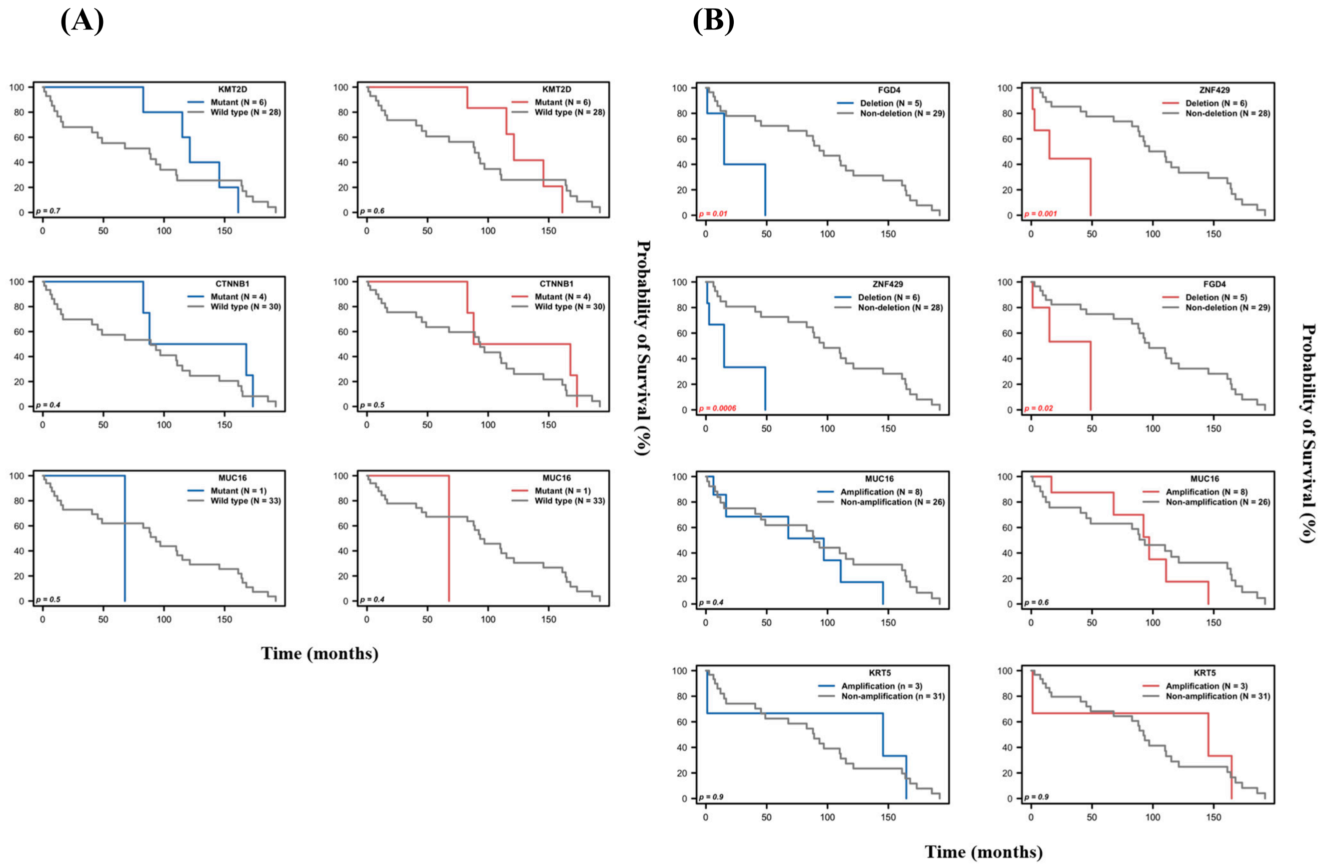

Supplementary Figure S1: Kaplan–Meier analyses of disease-free survival (DFS) and overall survival (OS) for cancer-associated genes among the 15 most frequently observed somatic mutations and CNV profiles in HB. **(A)** DFS (blue) and OS (red) of 34 HB patients with or without mutations in *KMT2D*, *CTNNB1*, and *MUC16*. **(B)** DFS (blue) and OS (red) of patients with or without CNV alterations, including deletions in *FGD4* and *ZNF429* and amplifications in *MUC16* and *KRT5*. Significance was determined by the log-rank test.

Supplementary Table S1: The clinical information of the patients selected to create the PoN.

Run accession	Age (y)	Gender
SRR17485390	26	female
SRR17485345	70	male
SRR17485340	83	male
SRR17485322	45	female
SRR17485295	65	male
SRR17485318	61	male
SRR17485292	52	female
SRR17485325	60	male
SRR17485405	50	male
SRR17485331	71	male
SRR17485290	63	male
SRR17485383	61	female
SRR17485307	70	male
SRR17485320	70	male
SRR17485350	45	male
SRR17485284	61	male
SRR17485336	66	male
SRR17485427	65	male
SRR17485384	67	male
SRR17485358	72	male
SRR17485283	51	female
SRR17485277	58	male
SRR17485297	66	male
SRR17485288	73	male
SRR17485334	62	male

Run accession	Age (y)	Gender
SRR17485329	64	male
SRR17485302	56	male
SRR17485296	62	male
SRR17485304	84	female
SRR17485274	65	male
SRR17485343	43	male
SRR17485337	69	male
SRR17485303	64	male
SRR17485305	70	male
SRR17485286	61	male
SRR17485293	62	male
SRR17485282	54	female
SRR17485294	54	male
SRR17485301	72	male
SRR17485287	63	female
SRR17485381	73	male
SRR17485280	52	male
SRR17485400	45	female
SRR17485291	77	female
SRR17485279	66	female
SRR17485276	57	male
SRR17485275	56	female
SRR17485289	77	female
SRR17485319	68	male
SRR17485281	57	male

Supplementary Table S2: List of our customized cancer-associated genes.

Gene Symbol	Gene Symbol	Gene Symbol	Gene Symbol	Gene Symbol
AADACL4	*ARHGAP26*	*BCL11A*	*C11orf93*	*CD79A*
ABCA4	*ARHGEF12*	*BCL11B*	*C17orf68*	*CD79B*
ABCD1	*ARID1A*	*BCL2*	*C2orf44*	*CDC25A*
ABI1	*ARID1B*	*BCL2L11*	*CACNA1D*	*CDC73*
ABL1	*ARID2*	*BCL3*	*CALR*	*CDH1*
ABL2	*ARID5B*	*BCL6*	*CAMTA1*	*CDH11*
ACD	*ARNT*	*BCL7A*	*CANT1*	*CDK12*
ACKR3	*ASPSCR1*	*BCL9*	*CARD11*	*CDK4*
ACSL3	*ASXL1*	*BCL9L*	*CARS*	*CDK6*
ACSL6	*ASXL2*	*BCOR*	*CASC5*	*CDKN1A*
ACVR1	*ATF7IP*	*BCORL1*	*CASP10*	*CDKN1B*
ACVR2A	*ATIC*	*BCR*	*CASP8*	*CDKN1C*
AFF1	*ATM*	*BIRC3*	*CBFA2T3*	*CDKN2A*
AFF3	*ATP1A1*	*BLM*	*CBFB*	*CDKN2C*
AFF4	*ATP2B3*	*BMP4*	*CBL*	*CDX2*
AIP	*ATR*	*BMPR1A*	*CBLB*	*CEBPA*
AKAP9	*ATRX*	*BRAF*	*CBLC*	*CHCHD7*
AKT1	*AXIN1*	*BRCA1*	*CCDC6*	*CHD4*
AKT2	*AXIN2*	*BRCA2*	*CCNB1IP1*	*CHEK2*
ALDH2	*B2M*	*BRD3*	*CCND1*	*CHIC2*
ALK	*BAP1*	*BRD4*	*CCND2*	*CHN1*
AMER1	*BARD1*	*BRIP1*	*CCND3*	*CIC*
APC	*BAX*	*BTG1*	*CCNE1*	*CIITA*
APOB	*BAZ1A*	*BTK*	*CD274*	*CLP1*
AR	*BCL10*	*BUB1B*	*CD74*	*CLTC*
CLTCL1	*DIP2B*	*ERCC8*	*FCGR2B*	*GATA2*
CNBP	*DIS3L2*	*ERG*	*FCRL4*	*GATA3*
CNOT3	*DKC1*	*ESR1*	*FEV*	*GMPS*
CNTRL	*DNM2*	*ETNK1*	*FGFR1*	*GNA11*
COL1A1	*DNMT3A*	*ETV1*	*FGFR1OP*	*GNAQ*
COL2A1	*DROSHA*	*ETV4*	*FGFR2*	*GNAS*
COX6C	*DUSP10*	*ETV5*	*FGFR3*	*GNB1*
CREB1	*DUX4L1*	*ETV6*	*FGFR4*	*GOLGA5*
CREB3L1	*EBF1*	*EWSR1*	*FH*	*GOPC*
CREB3L2	*ECT2L*	*EXT1*	*FHIT*	*GPC3*
CREBBP	*EGFR*	*EXT2*	*FIP1L1*	*GPHN*

Supplementary Table S2: List of our customized cancer-associated genes (*Cont*).

Gene Symbol	Gene Symbol	Gene Symbol	Gene Symbol	Gene Symbol
CRLF2	*EIF3H*	*EZH2*	*FLCN*	*GREM1*
CRTC1	*EIF4A2*	*EZR*	*FLG*	*GRIN2A*
CRTC3	*ELANE*	*FAM175A*	*FLI1*	*H3F3A*
CTCF	*ELF4*	*FAM46C*	*FLNA*	*H3F3B*
CTNNB1	*ELK4*	*FANCA*	*FLT3*	*HABP2*
CTR9	*ELL*	*FANCB*	*FMR1*	*HAX1*
CUX1	*ELN*	*FANCC*	*FNBP1*	*HDAC2*
CXCR4	*EML4*	*FANCD2*	*FOXA1*	*HDAC7*
CYLD	*EP300*	*FANCE*	*FOXL2*	*HERPUD1*
DAXX	*EPAS1*	*FANCF*	*FOXO1*	*HEY1*
DDB2	*EPCAM*	*FANCG*	*FOXO3*	*HIP1*
DDIT3	*EPO*	*FANCI*	*FOXO4*	*HIST1H3B*
DDR2	*EPS15*	*FANCL*	*FOXP1*	*HIST1H4I*
DDX10	*ERBB2*	*FANCM*	*FRG1*	*HLA-A*
DDX3X	*ERBB3*	*FAP*	*FRG2*	*HLF*
DDX41	*ERBB4*	*FAT1*	*FSTL3*	*HMGA1*
DDX5	*ERCC1*	*FAT2*	*FUBP1*	*HMGA2*
DDX6	*ERCC2*	*FAT4*	*FUS*	*HMGN2P46*
DEK	*ERCC3*	*FBN2*	*G6PC3*	*HNF1A*
DGCR8	*ERCC4*	*FBXO11*	*GAR1*	*HNRNPA2B1*
DHX15	*ERCC5*	*FBXO28*	*GAS7*	*HOOK3*
DICER1	*ERCC6*	*FBXW7*	*GATA1*	*HOXA11*
HOXA13	*KAT6A*	*LZTR1*	*MNX1*	*NFKBIE*
HOXA9	*KAT6B*	*MAF*	*MPL*	*NHP2*
HOXB13	*KBTBD4*	*MAFB*	*MRE11A*	*NIN*
HOXC11	*KCNJ5*	*MALAT1*	*MSH2*	*NIPBL*
HOXC13	*KDM5A*	*MALT1*	*MSH6*	*NKX2-1*
HOXD11	*KDM5C*	*MAML2*	*MSI2*	*NONO*
HOXD13	*KDM6A*	*MAP2K1*	*MSN*	*NOP10*
HRAS	*KDR*	*MAP2K2*	*MTCP1*	*NOTCH1*
HSP90AA1	*KDSR*	*MAP2K4*	*MTOR*	*NOTCH2*
HSP90AB1	*KEAP1*	*MAP3K1*	*MUC1*	*NPAT*
ID3	*KIAA1549*	*MAPK1*	*MUTYH*	*NPM1*
IDH1	*KIF5B*	*MAX*	*MYB*	*NR4A3*
IDH2	*KIT*	*MC1R*	*MYC*	*NRAS*
IGF2R	*KLF4*	*MDM2*	*MYCL*	*NSD1*
IGH	*KLF6*	*MDM4*	*MYCN*	*NT5C2*

Supplementary Table S2: List of our customized cancer-associated genes (*Cont*).

Gene Symbol
IGK
IGL
IKBKB
IKZF1
IKZF3
IL2
IL21R
IL6ST
IL7R
IRF4
IRS4
ITK
JAK1
JAK2
JAK3
JAZF1
JMJD1C
JUN
PAX7
PAX8
PBRM1
PBX1
PCBP1
PCM1
PCSK7
PDCD1LG2
PDE4DIP
PDGFB
PDGFRA
PDGFRB
PER1
PHF6
PHOX2B
PICALM
PIK3CA
PIK3CB
PIK3R1

Gene Symbol
KLK2
KMT2A
KMT2C
KMT2D
KRAS
KTN1
LAMA5
LASP1
LCK
LCP1
LEF1
LHFP
LIFR
LMO1
LMO2
LPP
LRIG3
LYL1
POU2AF1
POU5F1
PPARG
PPM1D
PPP2R1A
PPP6C
PRCC
PRDM1
PRDM16
PRDM9
PREX2
PRF1
PRKACA
PRKAR1A
PRRX1
PSIP1
PTCH1
PTCH2
PTCHD4

Gene Symbol
MDS2
MECOM
MED12
MEN1
MET
MFN2
MGA
MITF
MKL1
MLF1
MLH1
MLLT1
MLLT10
MLLT11
MLLT3
MLLT4
MLLT6
MN1
RAD51C
RAD51D
RAF1
RAG1
RAG2
RALGDS
RANBP17
RANBP2
RAP1GDS1
RARA
RB1
RBM10
RBM15
RBM8A
RECQL
RECQL4
REL
REST
RET

Gene Symbol
MYD88
MYH11
MYNN
MYOD1
NACA
NBN
NCKIPSD
NCOA1
NCOA2
NCOA4
NCOR1
NCOR2
NDRG1
NF1
NF2
NFE2L2
NFIB
NFKB2
RPL26
RPL35A
RPL5
RPN1
RPS10
RPS17
RPS19
RPS24
RPS26
RPS7
RTEL1
RUNX1
SALL4
SAMD9
SAMD9L
SBDS
SCG5
SCN9A
SDC4

Gene Symbol
NTHL1
NTRK1
NTRK3
NUMA1
NUP214
NUP98
NUTM1
NUTM2A
NUTM2B
OLIG2
OMD
OTX2
P2RY8
PALB2
PALLD
PATZ1
PAX3
PAX5
SH2B3
SH2D1A
SH3GL1
SHOC2
SHROOM2
SI
SIRPA
SIX1
SIX2
SLC34A2
SLC45A3
SLX4
SMAD2
SMAD3
SMAD4
SMAD7
SMARCA4
SMARCB1
SMARCE1

Supplementary Table S2: List of our customized cancer-associated genes (*Cont*).

Gene Symbol	Gene Symbol	Gene Symbol	Gene Symbol	Gene Symbol
PIM1	*PTEN*	*RHBDF2*	*SDHA*	*SMC1A*
PINK1	*PTPN11*	*RHOA*	*SDHAF2*	*SMC3*
PLAG1	*PTPN13*	*RHOH*	*SDHB*	*SMG8*
PLCG1	*PTPRB*	*RHPN2*	*SDHC*	*SMO*
PMEL	*PTPRD*	*RIT1*	*SDHD*	*SNX29*
PML	*PTPRK*	*RMI2*	*SELP*	*SOCS1*
PMS1	*PTPRT*	*RNF213*	*SEPT5*	*SOS1*
PMS2	*QKI*	*RNF43*	*SEPT6*	*SOX2*
POLD1	*RABEP1*	*ROS1*	*SEPT9*	*SPECC1*
POLD3	*RAC1*	*RPL10*	*SET*	*SPEN*
POLE	*RAD21*	*RPL11*	*SETBP1*	*SPOP*
POLH	*RAD50*	*RPL15*	*SETD2*	*SPRED1*
POLQ	*RAD51*	*RPL19*	*SF3B1*	*SPRTN*
POT1	*RAD51B*	*RPL22*	*SFPQ*	*SRC*
SRGAP3	*TET1*	*TRIP11*	*ZBTB16*	*CASP3*
SRSF2	*TET2*	*TRRAP*	*ZBTB7A*	*CASP9*
SRSF3	*TFAP4*	*TSC1*	*ZEB2*	*CCNC*
SS18	*TFE3*	*TSC2*	*ZFHX3*	*CCR4*
SS18L1	*TFG*	*TSHR*	*ZFP36L2*	*CCR7*
SSX1	*TFPT*	*TSPYL2*	*ZIC1*	*CD209*
SSX2	*TFRC*	*TTL*	*ZMIZ1*	*CD28*
SSX4	*TGFBR1*	*TYK2*	*ZMYM2*	*CDH10*
STAG2	*TGFBR2*	*U2AF1*	*ZMYM3*	*CDH17*
STAT3	*THRAP3*	*UBA2*	*ZNF217*	*CEP89*
STAT6	*TIAM1*	*UBR5*	*ZNF331*	*CHD2*
STK11	*TINF2*	*UNC13D*	*ZNF384*	*CHST11*
STX11	*TLX1*	*USP6*	*ZNF521*	*CLIP1*
STXBP2	*TLX3*	*USP7*	*ZRSR2*	*CNBD1*
SUFU	*TMEM127*	*USP8*	*A1CF*	*CNTNAP2*
SUZ12	*TMPRSS2*	*USP9X*	*ACVR1B*	*COL3A1*
SYK	*TNFAIP3*	*VHL*	*AFDN*	*CPEB3*
TAF15	*TNFRSF14*	*VTI1A*	*AKT3*	*CRNKL1*
TAL1	*TNFRSF17*	*WAS*	*ANK1*	*CSF1R*
TAL2	*TOP1*	*WDR64*	*APOBEC3B*	*CSF3R*
TARBP2	*TP53*	*WHSC1*	*ARAF*	*CSMD3*

Supplementary Table S2: List of our customized cancer-associated genes (*Cont*).

Gene Symbol	Gene Symbol	Gene Symbol	Gene Symbol	Gene Symbol
TBL1XR1	*TP63*	*WIF1*	*ARHGAP35*	*CTNNA1*
TBR1	*TPM3*	*WRAP53*	*ARHGAP5*	*CTNNA2*
TBX3	*TPM4*	*WRN*	*ARHGEF10*	*CTNND1*
TCEA1	*TPR*	*WT1*	*ARHGEF10L*	*CTNND2*
TCF12	*TRA*	*WWTR1*	*ASPM*	*CUL3*
TCF3	*TRAF7*	*XBP1*	*ATF1*	*CYP2C8*
TCF7L1	*TRB*	*XPA*	*BCL2L12*	*CYSLTR2*
TCF7L2	*TRD*	*XPC*	*BCLAF1*	*DCAF12L2*
TCL1A	*TRG*	*XPO1*	*BIRC6*	*DCC*
TCL6	*TRIM24*	*XPO5*	*BMP5*	*DCTN1*
TERC	*TRIM27*	*XRCC2*	*BTG2*	*DNAJB1*
TERT	*TRIM33*	*YWHAE*	*C15orf65*	*EED*
EIF1AX	*HGF*	*MUC4*	*PRKD1*	*SMARCD1*
EIF3E	*HIF1A*	*MUC6*	*PRPF40B*	*SND1*
ELF3	*HNRNPA2B1*	*MYH9*	*PTK6*	*SOX21*
EPHA3	*IGF2BP2*	*MYO5A*	*PTPN6*	*STAG1*
EPHA7	*ISX*	*N4BP2*	*PTPRC*	*STAT5B*
ERC1	*ITGAV*	*NAB2*	*PWWP2A*	*STIL*
FADD	*KAT7*	*NBEA*	*RAD17*	*STRN*
FAM131B	*KNL1*	*NFATC2*	*RAP1B*	*SUB1*
FAM135B	*KNSTRN*	*NR2F2*	*RFWD3*	*TEC*
FAM47C	*LARP4B*	*NRG1*	*RGPD3*	*TENT5C*
FAS	*LATS1*	*NSD2*	*RGS7*	*TFEB*
FAT3	*LATS2*	*NSD3*	*ROBO2*	*TMSB4X*
FBLN2	*LEPROTL1*	*NTRK2*	*RRAS2*	*TNC*
FEN1	*LHFPL6*	*NUTM2D*	*RSPO2*	*TNRC18*
FES	*LMNA*	*PABPC1*	*RSPO3*	*USP44*
FKBP9	*LRP1B*	*PAFAH1B2*	*RUNX1T1*	*VAV1*
FLT4	*LSM14A*	*PIK3R2*	*RXRA*	*WDCP*
FOXR1	*LYN*	*PIK3R3*	*S100A7*	*WNK2*
GLI1	*MACC1*	*PLEC*	*SETD1B*	*YAP1*
GOLPH3	*MAP3K13*	*POLG*	*SETDB1*	*ZCCHC8*
GPC5	*MB21D2*	*POLR2A*	*SFRP4*	*ZEB1*
GRM3	*MGMT*	*PPFIBP1*	*SGK1*	*ZNF429*
GSK3B	*MRTFA*	*PRDM2*	*SHTN1*	*ZNF479*
GTF2I	*MUC16*	*PRKCB*	*SKI*	*ZNRF3*

Supplementary Table S3: The potential targeted drugs for the driver mutations in HB

Alterations	Amino acid change	Drugs	Diseases*	Response	Level of evidence#
NRAS	Q61R	EGFR mAb inhibitor (Cetuximab)	CRA	Resistant	A, B, C, D
		EGFR mAb inhibitor (Cetuximab), Chemotherapy	CRA	Resistant	B
		EGFR mAb inhibitor (Panitumumab + Cetuximab)	CRA	Resistant	A
		EGFR mAb inhibitor (Panitumumab)	CRA	Resistant	A
		BRAF inhibitors	CM	Resistant	C
		BRAF inhibitor (Vemurafenib)	CM	Resistant	B, D
		BRAF inhibitors (Dabrafenib, Vemurafenib)	CM	Resistant	B
		MEK inhibitors	LUAD, CM, AML, ALL	Responsive	B, D
		MEK inhibitor (Trametinib)	ECD, LCD, CM	Responsive	A, B
		MEK inhibitor (Cobimetinib)	ECD, LCH	Responsive	A
		MEK inhibitor (Selumetinib)	Thyroid carcinoma	Responsive	C
		MEK inhibitor (Binimetinib)	CM	Responsive	D
		MEK inhibitors +/- PI3K inhibitors	CRA	Responsive	D
		MEK inhibitors + PI3K inhibitors	Myeloma	Responsive	D
		MEK inhibitor + PI3K inhibitor (Trametinib + Omipalisib)	CM	Responsive	D
		MEK inhibitors + CDK4/6 inhibitor (Binimetinib + Ribociclib)	CM	Responsive	C
		MEK inhibitors + CDK4/6 inhibitors	CM	Responsive	C
		MEK inhibitors + Pan-TK inhibitor (MEK inhibitors + Sorafenib)	Hepatic carcinoma	Responsive	C
		Pan-RAF inhibitors	CM	Responsive	C
		ERK inhibitors	CM	Responsive	C
		HSP90 inhibitors	CM	Responsive	D
		Chemotherapy (Temozolomide)	CM	Responsive	C
CTNNB1	D32G, D32Y, I35S, T41A	Tankyrase inhibitors	CRA	Resistant	D

* CRA = Colorectal adenocarcinoma, CM = Cutaneous melanoma, LUAD = Lung adenocarcinoma, AML = Acute myeloid leukemia, ALL = Acute lymphoblastic leukemia, ECD = Erdheim-Chester disease, LCH = Langerhans cell histiocytosis

Level of evidence are obtained from the VICC integrated knowledge base and are classified as follow: Level A - corresponds to biomarkers used in professional guidelines of FDA approved drugs, Level B - groups biomarkers observed in clinical trial, Level C -corresponds to biomarkers identified from small group studies or case studies, and Level D - biomarkers have been identified in pre-clinical studies

Supplementary Table S4: CNV profiles at chromosome arm identified in 34 HB patients.

Arm	Genes within region	Frequency (n, %)	q-value
**Chromosome arms with copy number amplifications (5)**			
9p	621	24/34, 70.59%	0.0119
11p	1162	20/34, 58.82%	0.0084
20p	583	23/34, 67.65%	0.0153
20q	1092	19/34, 55.88%	0.0177
22q	1258	20/34, 58.82%	0.0087
**Chromosome arms with copy number deletions (6)**			
4q	1467	24/34, 70.59%	0.0072
7p	926	23/34, 67.65%	0.0256
7q	1717	21/34, 61.76%	0.0452
19p	1331	22/34, 64.71%	0.0003
19q	2402	22/34, 64.71%	0.0001
22p	2	20/34, 58.82%	0.0029

Supplementary Table S5: CNV profiles of cancer-associated genes in focal regions identified in 34 HB patients.

Cytoband	q-value	Peak regions (bp)	GISTIC genes within region	Cancer-associated genes
**Regions with copy number amplifications (18)**				
12p13.31	2.96×10^{-74}	chr12:9277592-9453262	4	
2q21.1	1.82×10^{-30}	chr2:131462329-131846682	9	
18p11.21	3.04×10^{-28}	chr18:14859432-20921675	3	
6p21.33	9.13×10^{-19}	chr6:31980664-32069169	7	
19p13.2	1.57×10^{-13}	chr19:8889805-8991462	1	MUC16
16p11.2	2.64×10^{-13}	chr16:29122731-29368940	1	
21q22.3	1.09×10^{-12}	chr21:44523935-44647398	12	
22q11.21	4.34×10^{-11}	chr22:20680552-20718843	3	
11q12.2	6.46×10^{-9}	chr11:61128985-61250819	4	
3p21.31	6.92×10^{-8}	chr3:49685241-49691193	2	
17p11.2	7.37×10^{-11}	chr17:18382741-18494366	5	
12q13.13	3.60×10^{-3}	chr12:52252223-52659568	17	KRT5
19q13.42	4.22×10^{-3}	chr19:54837669-54889836	8	
16q24.2	6.48×10^{-3}	chr16:88012370-88111294	2	
22q11.21	6.72×10^{-9}	chr22:18173764-18910282	12	
17p11.2	1.47×10^{-5}	chr17:20439481-20590002	3	
17q21.2	1.38×10^{-2}	chr17:40978145-41196784	25	
14q32.33	3.20×10^{-2}	chr14:105578512-105771415	2	

Supplementary Table S5: CNV profiles of cancer-associated genes in focal regions identified in 34 HB patients (*Cont*).

Cytoband	q-value	Peak regions (bp)	GISTIC genes within region	Cancer-associated genes
**Regions with copy number deletions (21)**				
10p11.21	4.42×10^{-36}	chr10:35793288-37311901	1	
20q11.1	4.42×10^{-36}	chr20:26206387-31267559	10	
11p11.12	1.45×10^{-29}	chr11:49805636-50283381	3	
19p12	2.08×10^{-18}	chr19:19764986-27791054	49	ZNF429
11q14.3	1.07×10^{-16}	chr11:89684173-89802722	1	
12p11.21	2.20×10^{-14}	chr12:31047704-38317099	17	FGD4
18p11.21	2.58×10^{-14}	chr18:14800377-14837157	1	
2q13	1.64×10^{-12}	chr2:111766973-111778776	1	
4p16.3	9.92×10^{-10}	chr4:248714-363842	2	
13q12.11	2.69×10^{-9}	chr13:19324281-19637643	3	
21q11.2	3.72×10^{-5}	chr21:9814708-13546374	7	
3q11.1	8.82×10^{-5}	chr3:89474383-93986508	1	
10q21.3	1.59×10^{-4}	chr10:68303749-68530864	4	
8q24.3	2.93×10^{-3}	chr8:144768696-145138636	12	
17q12	4.29×10^{-3}	chr17:38101394-38209320	9	
5q35.3	7.41×10^{-3}	chr5:180889090-181538259	19	
6p21.33	1.01×10^{-2}	chr6:30894169-31059016	7	
8p23.1	1.03×10^{-2}	chr8:7354507-7926571	14	
1q31.3	1.88×10^{-2}	chr1:196747113-196986632	4	
3p14.3	3.33×10^{-2}	chr3:57471494-57761465	6	
14q32.33	4.16×10^{-2}	chr14:105857327-107043718	10	

Supplementary Table S6: Association between clinical variables and deletion of *FGD4* and *ZNF429*.

Variables		*FGD4*			*ZNF429*		
		deletion	non-deletion	P-value	deletion	non-deletion	P-value
Age (years)	≤ 3	4 (80%)	24 (82.8%)	1.0000	4 (66.7%)	24 (85.7%)	0.6026
	> 3	1 (20%)	5 (17.2%)		2 (33.3%)	4 (14.3%)	
Gender	Male	5 (100%)	18 (62.1%)	0.2473	6 (100%)	17 (60.7%)	0.1658
	Female	0 (0%)	11 (37.9%)		0 (0%)	11 (39.3%)	
PRETEXT stage	II	0 (0%)	14 (48.3%)	0.1217	1 (16.7%)	13 (46.4%)	0.3865
	III	3 (60%)	10 (34.5%)		3 (50%)	10 (35.7%)	
	IV	2 (40%)	5 (17.2%)		2 (33.3%)	5 (17.9%)	
Risk stratification	Low	2 (40%)	4 (13.8%)	0.3481	2 (33.3%)	4 (14.3%)	0.5180
	Intermediate	1 (20%)	11 (37.9%)		2 (33.3%)	10 (35.7%)	
	High risk	2 (40%)	14 (48.3%)		2 (33.3%)	14 (50%)	
Pretreatment AFP	≤ 100 ng/ml	1 (20%)	2 (6.9%)	0.9200	1 (16.7%)	2 (7.1%)	1.0000
	> 1000 ng/ml	4 (80%)	27 (93.1%)		5 (83.3%)	26 (92.9%)	
